# Interleukin-17A mRNA Expression is Associated with the Prognosis of Patients with Colorectal Cancer: A Pooled Meta-Analysis

**DOI:** 10.5152/tjg.2022.211071

**Published:** 2022-12-01

**Authors:** Wei Yang, Jiejing Chen, Hailiang Liang, Wei Wu

**Affiliations:** 1 Department of General Surgery, Affiliated Hospital of Yangzhou University, Yangzhou, Jiangsu, China

**Keywords:** Colorectal cancer, IL-17A·mRNA, meta-analysis, prognosis

## Abstract

**Background:**

Interleukin-17A is a proinflammatory cytokine that is produced by TH17 cells, and plays a dual role in tumor progression, infectious diseases, and autoimmune disorders. Interleukin-17A is induced during colorectal tumorigenesis and angiogenesis, although some studies have reported an anti-tumor effect as well. The aim of our study was to assess the prognostic role of interleukin-17A in colorectal cancer and determine the potential mechanisms.

**Methods:**

The GEO database was searched using the keyword “colorectal cancer”, and 10 datasets were identified that included interleukin-17A mRNA expression and survival data of several colorectal cancer patient cohorts. The patients were stratified into the interleukin-17A^high^ and interleukin-17A^low^ groups based on the median expression level.

**Results:**

Higher interleukin-17A mRNA levels were associated with better overall survival rates and the early tumor stage, indicating a protective role of interleukin-17A in colorectal cancer. Furthermore, interleukin-17A mRNA expression also correlated positively with that of TNFS11, CCR6, and CCL20, indicating that the anti-tumor effect of interleukin-17A is likely mediated by enhancing tumor antigen presentation by dendritic cells and recruiting the activated tumor-specific CD8^+^ cytotoxic T lymphocytes. The IL-23 and STAT3 mRNA levels were also significantly higher in the interleukin-17A^high^ group, which points to an upstream regulatory role of IL-23/STAT3 axis. Finally, the immune checkpoints PDCD1 (PD-1) and CD274 (PDL-1) were also positively correlated with interleukin-17A mRNA expression, indicating that interleukin-17A is a promising predictor of the immunotherapeutic outcome of PD-1/PDL-1 blockade in colorectal cancer.

**Conclusion:**

Interleukin-17A mRNA is a protective factor in colorectal cancer and a promising biomarker for assessing the prognosis and immunotherapeutic response.

**Main Points**

- Higher interleukin-17A (IL-17A) mRNA levels were associated with better overall survival rates and the early tumor stage in colorectal cancer (CRC).
- Interleukin-17A mRNA expression correlated positively with that of TNFS11, CCR6, and CCL20, indicating that the anti-tumor effect of IL-17A is likely mediated by the activated tumor-specific CD8^+^ cytotoxic T lymphocytes.
- IL-23/STAT3 axis plays an upstream regulatory role of IL-17A.
- Interleukin-17A is a promising predictor of the immunotherapeutic outcome of PD-1/PDL-1 blockade in CRC.

## Introduction

Colorectal cancer (CRC) is a common malignancy of the digestive tract, and its incidence rate is steadily increasing worldwide. It is estimated to become the third most commonly diagnosed cancer in China.^1^ A greater understanding of the colorectal tumorigenic mechanisms and the development of individualized treatment strategies have improved clinical outcomes over the last 20 years. Multiple factors such as poor diet, old age, genetic landscape and somatic mutations have been identified that increase the risk of CRC.^2^ In addition, recent studies have also associated the tumor microenvironment (TME) with the progression and clinical outcome of CRC.^3^ The TME consists of tumor cells, stromal cells and the infiltrating immune cells that produce large amounts of cytokines with both pro- and anti-tumorigenic effects.^4^ Due to this highly complex and heterogenous immune landscape, CRC is often considered an immunogenic malignancy.^5,6^ Colorectal cancer cells evade elimination by the host immune system during the initial stages of tumor growth and invasion.^7^ Furthermore, the immune cells infiltrating into the TME aggravate tumor progression by promoting inflammation, stromal cell proliferation, vascularization and metastases.^8^ Although the rapid tumor growth elicits an immune response characterized by the recruitment and activation of CD8^+^ cytotoxic T lymphocytes (CTLs) that release granzyme B and interferon-gamma,^9^ some immune cells and cytokines in the TME demonstrate plasticity in terms of inhibiting and promoting tumor growth.^10^

Given the crucial role of immune cells and inflammatory factors in tumor development and progression, it is essential to identify cytokine biomarkers that can predict patient prognosis and therapeutic effect.^11^ IL-17 comprises of a family of 5 pro-inflammatory cytokines, including IL-17B, IL-17C, IL-17D, IL-17E, and IL-17F,^12^ that are mainly secreted by CD4+ T-helper (Th17) cells, natural killer cells, CD8+ T cells, neutrophils, macrophages and dendritic cells (DCs).^13,14^ Th17 cells are a major infiltrating immune cell type in tumors, and produce pro-tumorigenic cytokines like interlukin-17A (IL-17A).^15^ Interlukin-17A is regulated by several transcription factors such as NFATc1, ROR and STAT3 that are in turn activated by IL-23.^16-18^ Studies have reported high levels of IL-17A in the sera and tumor tissues of CRC patients, which correlate significantly with worse prognosis.^19^ It not only enhances CRC cell proliferation but also promotes angiogenesis by stimulating VEGF secretion from the tumor cells.^20^ In contrast, some studies have reported an anti-tumor role of IL-17A in CRC.^21,22^ Interlukin-17A activates chemokines like CCR6 and CCL20 that are known to recruit anti-tumor cytotoxic CD8+ T cells. In addition, TNFSF11 (RANKL)-mediated tumor antigen presentation by the DCs to cytotoxic CD8+ T cells is also dependent on IL-17A.^23^ Furthermore, Karabulut et al^24^ found no association between IL-17 levels and the overall survival of CRC patients. Therefore, the prognostic relevance of IL-17A in CRC remains to be established. To this end, we assessed the correlation between IL-17A mRNA expression and prognosis of CRC patients from GEO datasets. In addition, we also determined the possible mechanisms underlying the inhibitory effect of IL-17A, as well as its ability to predict the immunotherapy outcome in CRC.

## Materials and Methods

The relevant datasets in the GEO database were searched using the keyword “colorectal cancer” by 2 investigators independently. Any discrepancies were resolved following a discussion with the team.

The clinical information and IL-17A expression data were extracted from 10 datasets by 2 authors independently. The first author’s name, country or region, publication year, sample number, tumor stage, survival time, and survival status were recorded. The IL-17A mRNA expression and survival data were analyzed using the “GEO2R” program. The quality of the datasets was assessed according to the Newcastle-Ottawa Quality Assessment Scale (NOS), and the scores ranging from 6 to 9 indicated high-quality studies.

### Statistical Analysis

Interleukin-17A mRNA expression data were extracted and expressed as metric variables. The median cutoff was determined by Yoden Index according to the survival status, and the patients were stratified into the high expression and low expression groups. The survival curve was plotted using the Kaplan–Meier method. Hazard risks (HRs) and 95% CI were calculated using the Cox proportional hazards model to measure the prognostic effect of IL-17A mRNA. The heterogeneity of the pooled HR was evaluated by the Cochran *Q* and *I* ^2^ test. The random-effect model was used when *P* < .1 or *I* ^2^ > 50%, and the fixed-effect model was employed when *P* > .1 or *I* ^2^ < 50%. Publication bias was assessed by the Revman funnel chart. The weighted mean difference and 95% CI were used to express the effective level of measurement data. *P* < .05 was considered statistically significant.

## Results

### Study Characteristics

**Figure 1.**
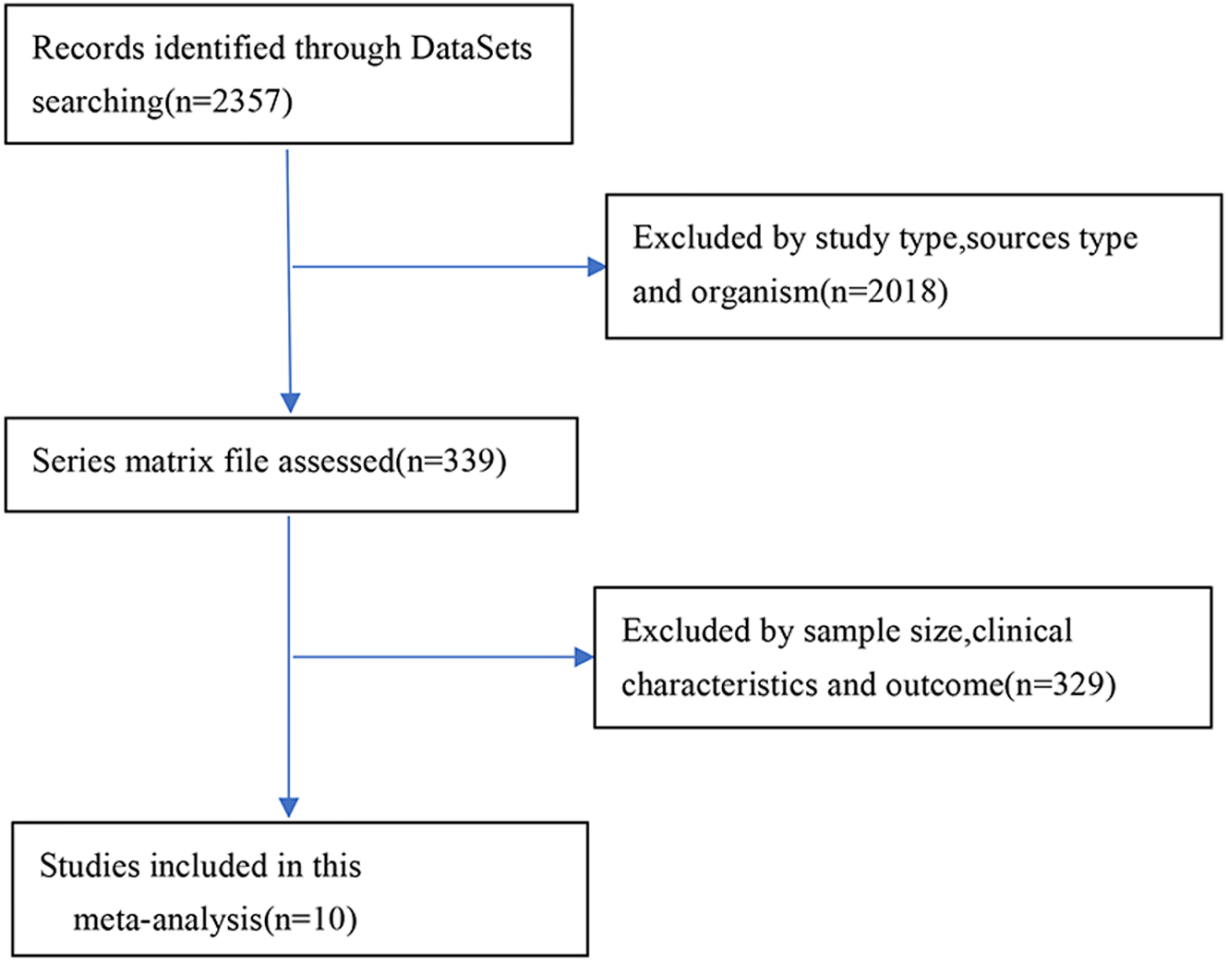
Flow diagram of the dataset selection process.

The initial search yielded 2357 relevant datasets, of which 2018 were retrieved after screening for the study type and organism. After analyzing the clinical characteristics and survival outcome, the GSE17538, GSE16125, GSE17536, GSE17537, GSE38832, GSE39582, GSE75500, GSE87211, GSE103479, and GSE71187 datasets were selected that met the inclusion criteria (Figure 1). The clinical characteristics of all included studies are summarized in Table 1. The studies were conducted across the United Kingdom, Italy, USA, France, China, and the Netherlands, and all reported overall survival and IL-17A mRNA expression. The NOS scores of the studies ranged from 6 to 9, indicating the high quality of the results.

### Overall Survival

**Figure 2.**
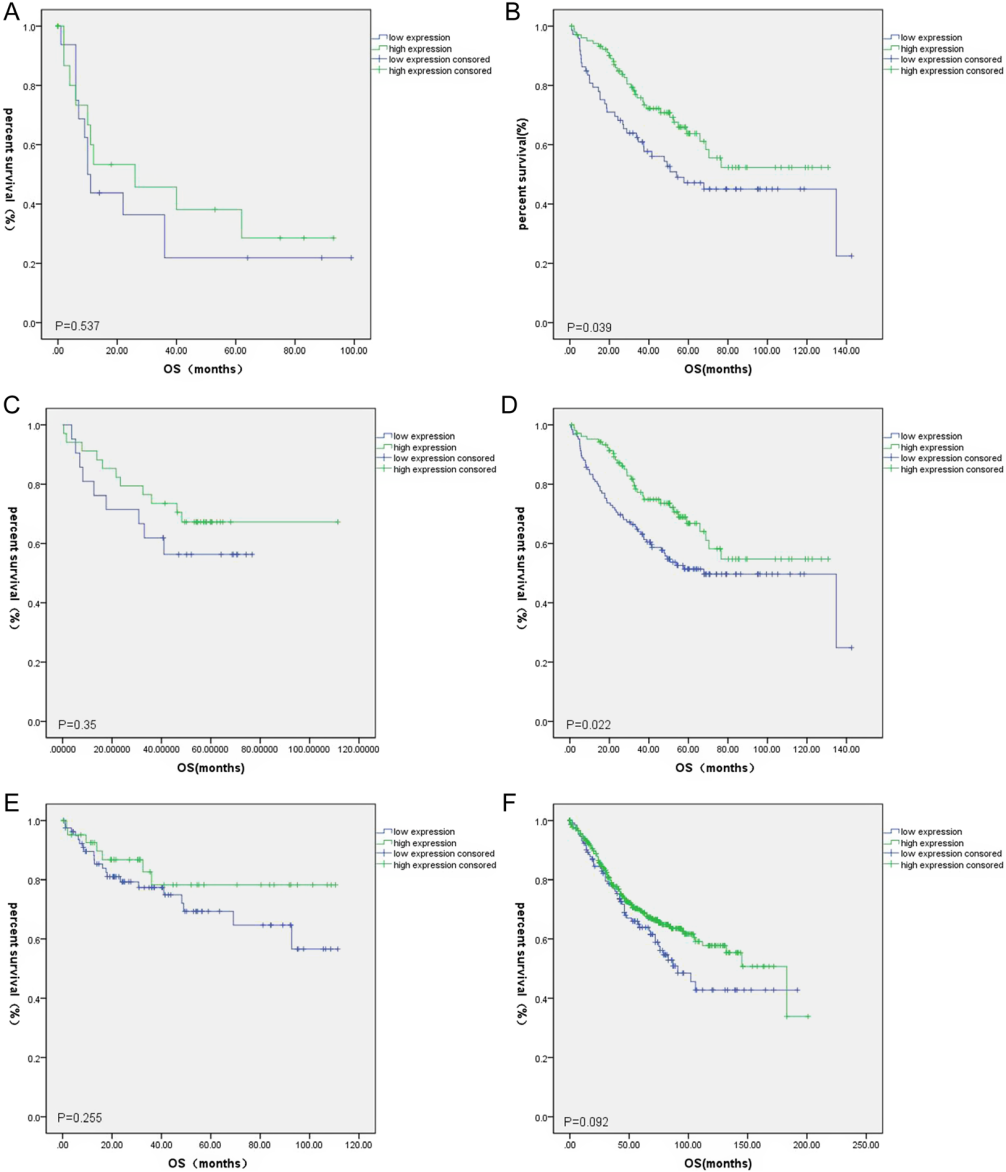
High mRNA expression of IL-17A in colorectal cancer patients was associated with a better prognosis. (A) Kaplan–Meier analysis of dataset GSE16125. (B) Kaplan–Meier analysis of dataset GSE17536. (C) Kaplan–Meier analysis of dataset GSE17537. (D) Kaplan–Meier analysis of dataset GSE17538. (E) Kaplan–Meier analysis of dataset GSE38832. (F) Kaplan–Meier analysis of dataset GSE39582. IL-7A, interleukin-17A.

**Figure 3.**
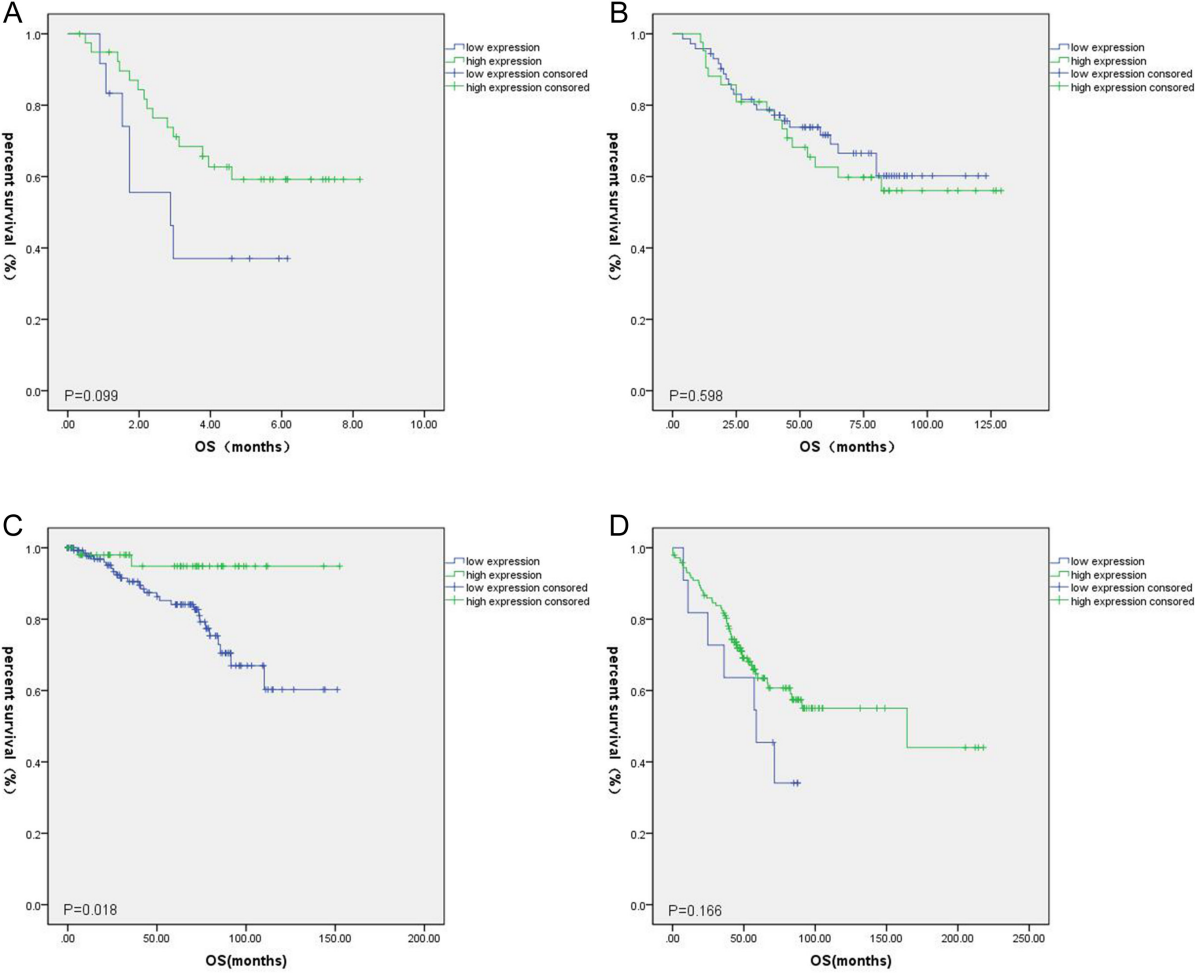
High mRNA expression of IL-17A in colorectal cancer patients was associated with a better prognosis. (A) Kaplan–Meier analysis of dataset GSE71187. (B) Kaplan–Meier analysis of dataset GSE75500. (C) Kaplan–Meier analysis of dataset GSE87211. (D) Kaplan–Meier analysis of dataset GSE103479. IL-7A, interleukin-17A.

**Figure 4.**
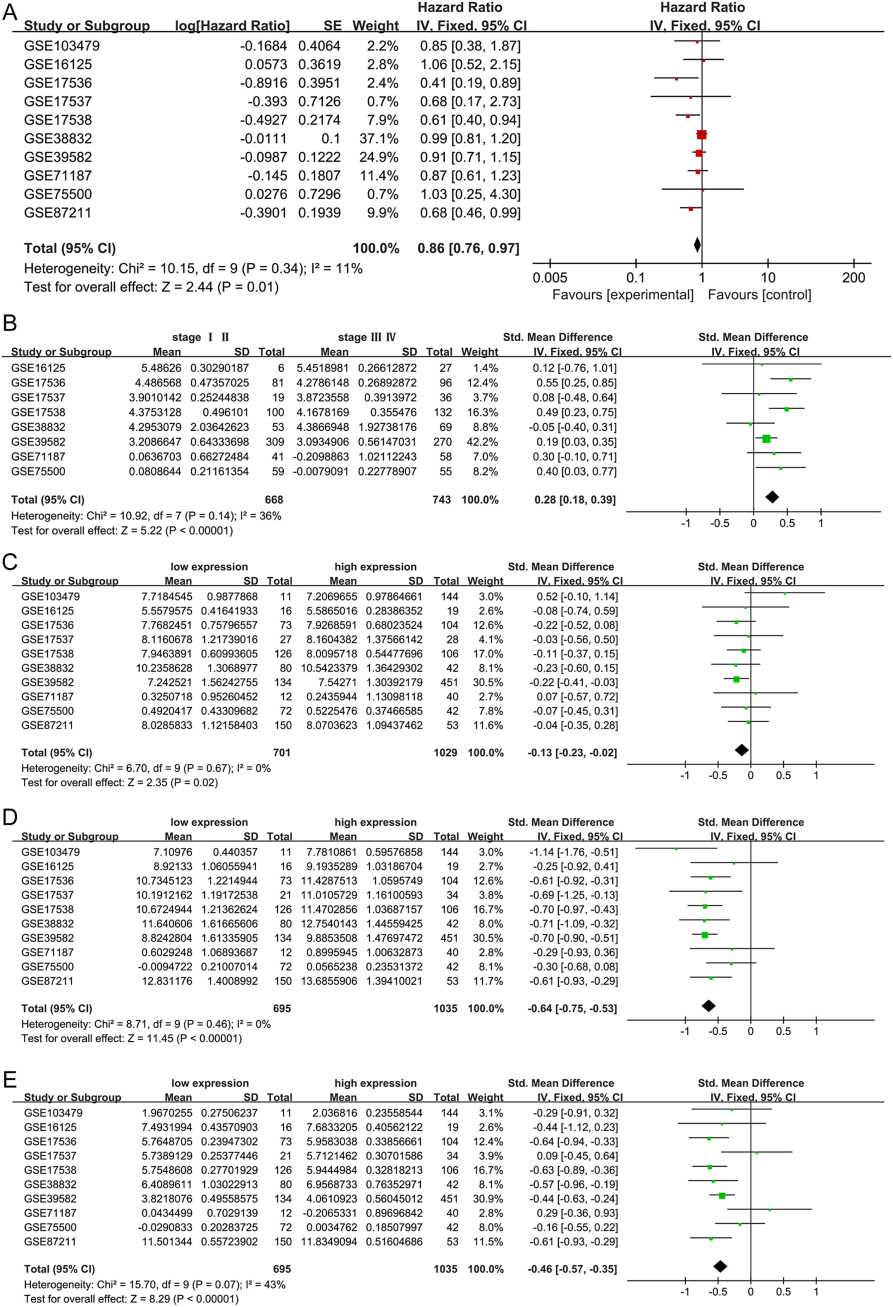
(A) Forest plots of studies evaluating hazard ratios of high IL-17A mRNA expression in colorectal cancer for overall survival. (B) IL-17A mRNA expression level between early stage and advanced stage in colorectal cancer. (C) TNFSF11 mRNA expression level between high IL-17A group and low IL-17A group. (D) CCL20 mRNA expression level between high IL-17A group and low IL-17A group. (E) CCR6 mRNA expression level between high IL-17A group and low IL-17A group. IL-7A, interleukin-17A.

The Kaplan–Meier curves of all 10 datasets are shown in Figures 2 and 3. Univariate analysis was performed on the survival status and duration, and IL-17A mRNA expression, and the forest plots of the HRs for overall survival (OS) are shown in Figure 4A. The fixed-effects model was used due to lack of significant heterogeneity (*I* ^2^ = 11%, *P *= .34). Higher expression of IL-17A mRNA was associated with longer OS (HR = 0.86; 95% CI = 0.76-0.97; *P *= .01). The sensitivity analysis was performed by excluding individual studies that did not significantly alter the results, indicating reliability. Finally, the Revman funnel plot did not show any significant publication bias (data not shown).

### Tumor Stage

Eight datasets included information on the tumor stage. The patients were stratified into early stages (I and II) and advanced stages (III and IV), and higher IL-17A mRNA levels correlated to early stage of the disease (SMD = 0.28; 95% CI = 0.18-0.39; *P *< .00001; Figure 4B). The fixed-effects model was used due to a lack of obvious statistical heterogeneity (*I* ^2^ = 36%, *P* = .14), sensitivity analysis indicated overall reliable results, and no significant publication bias was detected across the 8 studies (data not shown).

### Anti-tumor Mechanism

Ten datasets included the expression data of TNFSF11 and CCR6-CCL20 mRNAs, which mediate the anti-tumor effects of IL-17A. As shown in Figure 4C,4D,4E, higher IL-17A mRNA expression was associated with higher levels of TNFSF11 (SMD = −0.13; 95% CI = −0.23, −0.02; *P* = .02), CCL20 (SMD = −0.64; 95% CI = −0.75, −0.53; *P *< .00001) and CCR6 (SMD = −0.46; 95% CI = −0.57, −0.35; *P* < .00001). This strongly indicates that the anti-tumor activity of IL-17A in CRC is mediated by DCs expressing TNFS11 and the CCR6-CCL20 chemotactic axis, which induce the engagement of the CD8+ T cells. Sensitivity analysis indicated reliable results and no significant publication bias was detected (data not shown).

### Regulation of IL-17A

**Figure 5.**
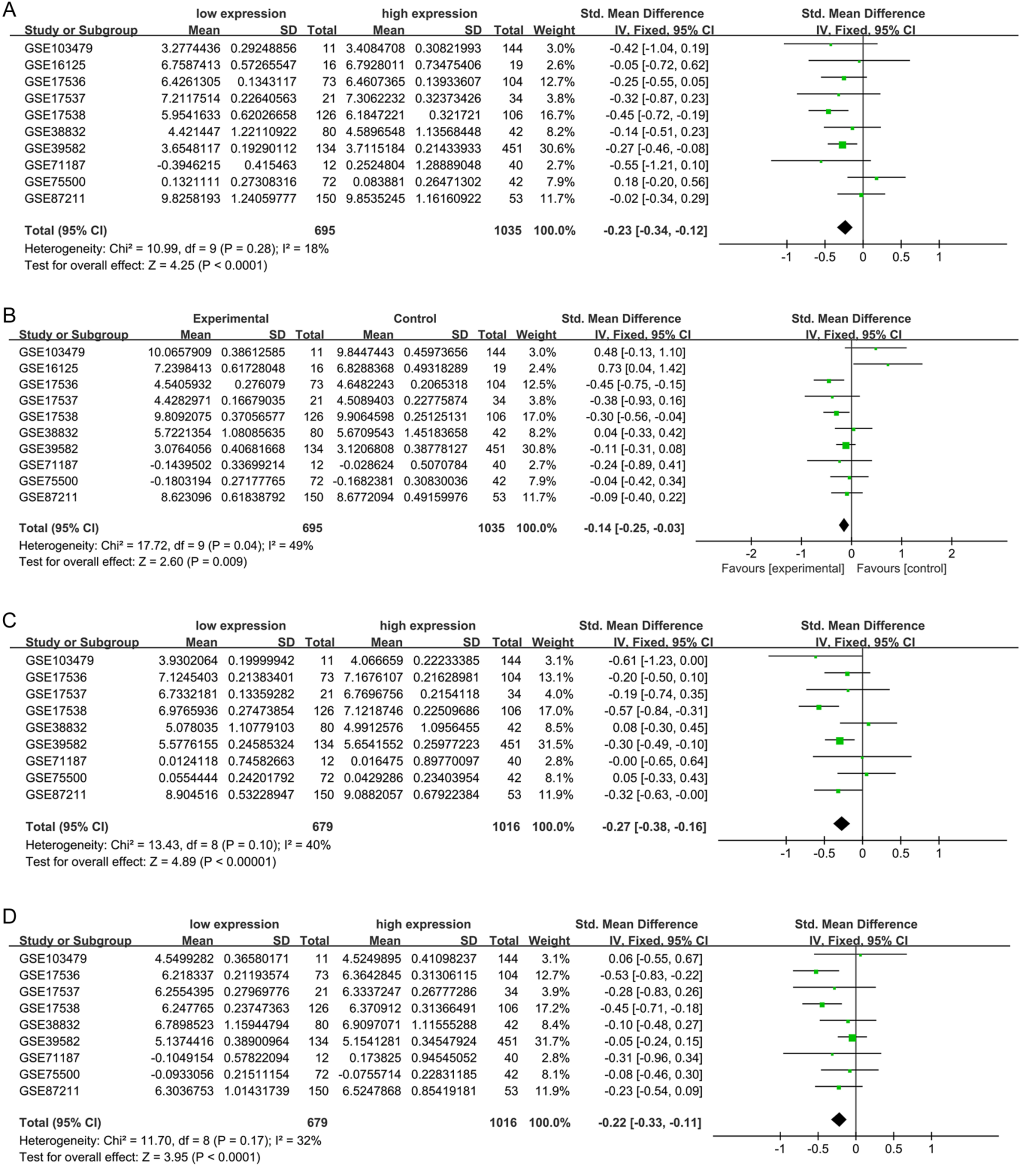
(A) IL-23 mRNA expression level between high IL-17A group and low IL-17A group. (B) STAT3 mRNA expression level between high IL-17A group and low IL-17A group. (C) PDCD1 mRNA expression level between high IL-17A group and low IL-17A group. (D) CD274 mRNA expression level between high IL-17A group and low IL-17A group. IL-7A, interleukin-17A.

IL-23 is essential for the differentiation of Th17 cells and IL-17 secretion. In addition, the IL-23/STAT3/IL-17 axis plays an important role in tumor progression and inflammation. Ten datasets included the data of IL-23/STAT3 expression, and higher IL-17A mRNA level was associated with higher levels of both IL-23 (SMD =−0.23; 95% CI = −0.34, −0.12; *P* < .0001) and STAT3 (SMD = −0.14; 95% CI = −0.25, −0.03; *P* = .009) mRNAs (Figure 5A and 5B) as per the fixed-effects model. The Revman funnel plot indicated a lack of any significant publication bias across the 10 studies (data not shown). Taken together, these findings suggest that IL-23 promotes IL-17 production in the CRC tissues via the STAT3 pathway.

### Association of IL-17A mRNA and Immunosuppression

Given the dynamic tumor immune microenvironment, IL-17A can elicit both cytotoxic and immunosuppressive effects**. **Therefore, we also analyzed the expression of the major immunosuppressive molecules PDCD1 (PD-1) and CD274 (PD-L1). Nine datasets included the data of PDCD1 and CD274 mRNA expression levels, which correlated significantly with that of IL-17A (PDCD1: SMD = −0.27; 95% CI = −0.38, −0.16; *P* < .00001; CD274: SMD = −0.22; 95% CI = −0.33, −0.11; *P* < .0001) as shown in the forest plots in Figure 5C and 5D. The fixed-effects model was used as *I* ^2^ < 50%. Revman funnel plot did not show any significant publication bias (data not shown). Previous studies show that overexpression of both PD-1 and PD-L1 is independent factors associated with better survival and response to immune checkpoint inhibitors (ICIs) in dMMR CRC. According to our findings, therefore, IL-17A mRNA level is a promising predictor of the response to anti-PD-1 and anti-PD-L1 immunotherapy.

## Discussion

Colorectal cancer is a multifactorial malignancy with complex mechanisms underlying its development and progression.^25^ The TME not only plays an important role in CRC initiation and progression but also the efficacy of chemotherapy and immunotherapy.^26,27^ Cytokines and chemokines mediate the interactions between the tumor cells and immediate microenvironment,^28^ and are therefore potential prognostic and therapeutic biomarkers of CRC and other tumors. The aim of our study was to assess the clinical relevance of IL-17A mRNA levels in CRC using data extracted from GEO datasets of several large patient cohorts.

Interleukin-17A is a pro-inflammatory cytokine that is associated with CRC progression, and regulates tumor cell growth and differentiation, as well as the tumor immune microenvironment.^12^ However, the predictive relevance of IL-17A in the clinical outcome and survival of CRC patients is ambiguous at present.^29^ Chen et al^30^ showed that high expression of IL-17A is a predictive marker of poor prognosis in CRC patients, whereas Tseng et al^31^ found that patients with higher serum levels of IL-17A had a shorter disease-free survival. Furthermore, Liu et al^20^ showed that IL-17 is associated with poor prognosis and promotes angiogenesis in colorectal tumors by stimulating VEGF secretion by the cancer cells. However, Lin et al^32^ reported better overall survival in patients with higher IL-17 expression. In our study as well, higher IL-17A mRNA levels were associated with greater overall survival rates in 10 patient cohorts, although the trend was not statistically significant in 7 cohorts. Sharp et al^33^ showed that advanced CRC stage was associated with elevated tumor levels of IL-17, whereas we found that higher IL-17A mRNA expression correlated to the early tumor stage. Thus, our findings point to a protective rather than a tumorigenic role of IL-17A in CRC. Our study is the first to investigate the association between IL-17A mRNA and CRC prognosis based on the data of patients from all tumor stages (except for the GSE103479 and GSE75500 datasets that included only stage II and III patients, and GSE87211 that did not include tumor stage data). In contrast, the study of Liu focused only on stage III patients.^20^

The aforementioned differences in prognostic relevance can be attributed to the distinct detection methods. All of the previous studies analyzed the in situ IL-17 protein expression by immunohistochemistry that characterized tissue localization as well. For instance, Amicarella et al^21^ found that an abundance of intraepithelial IL-17+ cells but not of stromal IL-17+ cells was associated with improved prognosis. Our findings on the other hand are based on the IL-17A mRNA levels in both tumor and stromal cells. Furthermore, the ethnic and geographical differences across the studies included in our meta-analysis may also contribute to the discrepancy.

The mechanisms underlying the anti-tumor effects of IL-17A are poorly understood. IL-17 promotes tumor invasion by increasing MMP-9 expression and VEGF-dependent angiogenesis,^19^ which is counterbalanced by the recruitment of CD16+ neutrophils and antigen-specific CTLs to the tumor tissues.^21^ This dual role of IL-17A in the microenvironment of CRC is not completely understood and maybe highly dynamic depending on the state of the adaptive immune system.^34^ For instance, we observed significantly higher levels of TNFSF11 (RANKL), CCR6 and CCL20 in the IL-17A^high^ versus IL-17A^low^ groups. RANKL and its cognate receptor RANK are expressed by distinct immune cells in the TME, especially the DCs,^35^ and present the tumor antigens to the CTLs. Our findings indicate that IL-17A induces CCL20 and CCR6 in the tumor tissues, which then recruit the tumor antigen-loaded DCs to activate the anti-tumor CD8+ T cells.^23^ RANKL also promotes the differentiation of the immature DCs to the active DCs and is therefore vital for antigen presentation and T cell activation.^36,37^ Taken together, IL-17A elicits an anti-tumor response by activating the CCL20-CCR6 and RANKL-RANK pathways.

IL-23 is secreted by tumor-associated immune cells and promotes IL-17 production^38^ along with IL-21, IL-6, and IL-1 via transcription factors such as NFATc1, ROR, and STAT3.^16^ Tang et al^39^ showed that IL-23 is required for the production of IL-17 by γδ T cells, and Richter et al^40^ affirmed its significant role during colon carcinogenesis. We found that both IL-23 and STAT3 mRNA expression levels were significantly higher in the IL-17A^high^ patient group, indicating that IL-17 is modulated by the IL-23/STAT3 axis. Finally, our findings also indicate that IL-17A upregulates immunosuppressive molecules including PDCD1 and CD274. Since PD-L1 expression in CRC tissues correlates positively with the efficacy of ICIs,^41^ patients expressing high levels of IL-17A mRNA in the tumors may benefit more from PDCD1 and CD274 inhibitors. Thus, IL-17 mRNA levels can predict the therapeutic outcome of PD-L1/PD-1 blockade.

In conclusion, high levels of IL-17A mRNA in the CRC tissues were associated with better survival and early tumor stage and likely indicate a favorable prognosis. However, since we only analyzed the IL-17A mRNA levels, and not the expression and localization of IL-17A protein, we cannot distinguish the function of tumor and stroma-induced IL-17. Further multicentric prospective studies are needed to verify the prognostic value of IL-17A in CRC patients.

## Figures and Tables

**Table 1.** Characteristics of Datasets for Pooled Meta-Analysis

GEO Datasets	Author	Year	Country	Sample Size	Stage	Outcome Measures	Quality Score
GSE103479	Allen W	2017	United Kingdom	156	II/III(84/72)	OS	8
GSE16125	Reid JF	2009	Italy	36	I/II/III/IV(1/5/8/19)	OS	6
GSE17536	Smith JJ	2009	USA	177	I/II/III/IV(24/57/57/39)	OS	8
GSE17537	Smith JJ	2009	USA	55	I/II/III/IV(4/15/19/17)	OS	6
GSE17538	Smith JJ	2009	USA	244	I/II/III/IV(24/57/57/39)	OS	8
GSE38832	Beauchamp RD	2014	USA	122	I/II/III/IV(18/35/39/30)	OS	7
GSE39582	Marisa L	2013	France	585	I/II/III/IV(38/271/210/60)	OS	9
GSE71187	Ning A	2017	China	189	I/II/III/IV(13/28/52/6)	OS	8
GSE75500	van den Broek E	2016	Netherlands	114	II/III(57/57)	OS	8
GSE87211	Hu Y	2017	USA	363	NR	OS	9

NR, not report; OS, overall survival.
